# Identification of carbapenem-resistant organism (CRO) contamination of in-room sinks in intensive care units in a new hospital bed tower

**DOI:** 10.1017/ice.2023.289

**Published:** 2024-03

**Authors:** Bobby G. Warren, Becky A. Smith, Aaron Barrett, Amanda M. Graves, Alicia Nelson, Erin Gettler, Sarah S. Lewis, Deverick J. Anderson

**Affiliations:** 1 Division of Infectious Diseases, Duke Center for Antimicrobial Stewardship and Infection Prevention, Durham, North Carolina; 2 Disinfection, Resistance and Transmission Epidemiology (DiRTE) Lab, Duke University School of Medicine, Durham, North Carolina; 3 Division of Infectious Diseases, Duke University School of Medicine, Durham, North Carolina

## Abstract

**Background:**

The origins and timing of inpatient room sink contamination with carbapenem-resistant organisms (CROs) are poorly understood.

**Methods:**

We performed a prospective observational study to describe the timing, rate, and frequency of CRO contamination of in-room handwashing sinks in 2 intensive care units (ICU) in a newly constructed hospital bed tower. Study units, A and B, were opened to patient care in succession. The patients in unit A were moved to a new unit in the same bed tower, unit B. Each unit was similarly designed with 26 rooms and in-room sinks. Microbiological samples were taken every 4 weeks from 3 locations from each study sink: the top of the bowl, the drain cover, and the p-trap. The primary outcome was sink conversion events (SCEs), defined as CRO contamination of a sink in which CRO had not previously been detected.

**Results:**

Sink samples were obtained 22 times from September 2020 to June 2022, giving 1,638 total environmental cultures. In total, 2,814 patients were admitted to study units while sink sampling occurred. We observed 35 SCEs (73%) overall; 9 sinks (41%) in unit A became contaminated with CRO by month 10, and all 26 sinks became contaminated in unit B by month 7. Overall, 299 CRO isolates were recovered; the most common species were *Enterobacter cloacae* and *Pseudomonas aeruginosa*.

**Conclusion:**

CRO contamination of sinks in 2 newly constructed ICUs was rapid and cumulative. Our findings support in-room sinks as reservoirs of CRO and emphasize the need for prevention strategies to mitigate contamination of hands and surfaces from CRO-colonized sinks.

More than 700,000 healthcare–associated infections (HAIs) occur each year in the United States.^1,2^ Pathogenic organisms that cause HAI can be transmitted numerous ways. Although these organisms were traditionally thought to be transmitted via healthcare providers’ hands, the hospital environment has emerged as a key source of transmission as well. In particular, transmission and outbreaks related to hospital wastewater sources are increasingly recognized.^3^ Among different water sources within the hospital, sinks located in patient rooms have been increasingly implicated as sources and reservoirs of epidemiologically important pathogens (EIPs).^4^ In fact, numerous outbreak investigations have implicated hand washing sinks and/or actions completed near sinks as the source of transmission.^5–13^ Most importantly, sinks are routinely contaminated with carbapenem-resistant organisms (CROs) such as carbapenem-resistant Enterobacterales (CREs). Outbreaks of CRE related to sink contamination have been well described.^3,14^

HAIs caused by CROs are particularly devastating, with high associated rates of mortality and considerable healthcare costs.^2^ Thus, understanding CRO sources and transmission dynamics are critical for prevention strategies. However, the origins and timing of sink contamination with CROs are not completely understood. We performed this prospective observational study to describe the timing, rate, and frequency of CRO contamination of in-room handwashing sinks in 2 intensive care units (ICUs) in a newly constructed hospital bed tower.

## Methods

### Study setting and design

We performed a prospective observational study of inpatient in-room handwashing sinks at Duke University Hospital (DUH) in Durham, North Carolina. Our primary objective was to evaluate CRO contamination of hospital in-room sinks in 2 neurological ICUs in a recently constructed bed tower. The key components of our study objective were to describe the timing and frequency of new CRO contamination compared to baseline measurements obtained prior to any patient occupying the room.

Unit A was opened to patients in July 2020, the first study samples were taken in September 2020, and the final samples were collected in May 2021 (n = 10 sampling episodes). The patient population and clinical service of unit A were moved to a new unit in the same bed tower, unit B, on June 6, 2021. The first study samples for unit B were obtained prior to this change, on June 3, 2021. Samples were obtained from unit B for the next year; however, 1 sampling episode was not completed due to supply shortages (n = 12 sampling episodes). Overall, the study period lasted from September 14, 2020, to June 6, 2022, with 22 total sampling episodes. Each unit was similarly designed with 26 rooms and in-room sinks available for inclusion in the study, resulting in sampling of 52 unique sinks during the study period. Patient data were collected retrospectively using a limited data set retrieved from electronic health records.

### Patient consent statement

This study was designated as exempt research by the Duke University Health System Institutional Review Board.

### Patient CRE surveillance procedures

In both study units, the Duke University Hospital Infection Prevention Department completed weekly point-prevalence CRE screening on Wednesday mornings via rectal swabs. Notably, patients were not rescreened (1) if a patient was admitted to the unit after orders were submitted, they were not tested, and (2) if patients were previously identified as CRE positive. These samples were processed by the hospital clinical microbiology laboratory and were not a part of our study activities.

### Study procedures

Environmental microbiological samples were taken every 4 weeks over the study period. Samples were obtained from 3 locations from each study sink: the top of the bowl, the drain cover (sieve shaped), and the p-trap. The sample from the top of the bowl included the horizontal surface surrounding the sink bowl as well as the sink handles. The sample from the drain cover included the outer exposed portion of the drain cover. The sample from the p-trap included an agitated liquid sample from the p-trap fluid. Sampling protocols were systematically used to ensure that the same locations and surface areas were cultured each time.

Routine environmental disinfection was performed in all study rooms according to standard hospital protocols. However, adherence was not measured. All rooms were single-patient rooms with no shared bathrooms. Routine disinfection was defined as (1) daily disinfection of patient-room surfaces with nonbleach solutions and (2) terminal disinfection of patient-room surfaces with nonbleach solutions and ultraviolet C (UV-C) treatment. Enhanced terminal disinfection was defined as routine terminal disinfection while substituting bleach solutions for nonbleach solutions. Environmental services employees were blinded to the study sampling strategy. Microbiological methods are detailed in the Supplementary Methods (online).

### Outcomes and analysis

The primary outcome was sink conversion events (SCEs), defined as newly identified CRO contamination of a sink from any of the 3 samples in which CRO had not previously been identified. Our secondary outcomes were (1) cumulative SCEs per unit, (2) time to SCEs, and (3) descriptive epidemiology regarding pathogen type, carbapenemase-producing CRE, (CP-CRE) and patient data of patients treated in study units. Study data were summarized using descriptive statistics, the χ^2^ test for categorical variables, and *t* tests for continuous variables, including means (±SD) and median (interquartile range [IQR]), as appropriate. *P* < .05 was considered significant. All statistical tests were 2-tailed and were performed using R software (R Foundation for Statistical Computing, Vienna, Austria).

### Secondary analysis methods

While completing the primary study, an epidemiological relevant situation regarding sinks arose, so a secondary analysis was completed. The detailed methods of this analysis are included in the Supplementary Methods (online).

## Results

Monthly sink samples were obtained 22 times during the study period, resulting in 1,638 total environmental cultures (780 from unit A and 858 from unit B). In total, 2,814 patients were admitted to study units while sink sampling occurred (Table 1). Overall, 857 patients (30%) underwent surveillance testing for CRE: 421 (28%) of 1,490 on unit A and 436 (33%) of 1,324 on unit B. Only 2 of these patients were identified as CRE positive (both with *Klebsiella pneumoniae* carbapenemase (KPC)–producing isolates) during the study period, and both were housed in unit A.

**Table 1.** Patient and Unit Characteristics in 2 Neurological ICUs in a Recently Constructed Bed Tower

Characteristic	Overall (N=2,814), No. (%)
**Patient characteristics**	
Age, median y (IQR)	61 (47–71)
**Sex**	
Female	1,360 (48)
Male	1,440 (51)
Not REPORTED/Unknown	14 (1)
**Race**	
White	1,674 (59)
Black or African American	885 (31)
Asian	61 (22)
Other	112 (4)
Not reported/Unknown	82 (3)
Study patients screened for CRE, total	857 (30)
**Unit characteristics**	
Length of stay, median d (IQR)	6 (3–15)
Positive bacteriological cultures, total	4,588
Blood	630 (14)
Urine	772 (17)
Tissue	499 (11)
Respiratory	123 (3)
Cerebrospinal fluid	725 (16)
Other	1,839 (40)
Positive CRE surveillance cultures, total	2

Note. ICU, intensive care unit; IQR, interquartile range; CRE, carbapenem-resistant Enterobacterales.

### SCEs

Our first samples from unit A were obtained ∼60 days after the unit was opened to patients. Of the 26 sinks, 4 were already contaminated with CRO and 3 were contaminated with CP-CRE upon initial evaluation in this unit and were only included in descriptive analyses. In contrast, all samples in unit B were obtained prior to patients being admitted to this unit; none grew CRO or CP-CRE at baseline.

**Figure 1.**
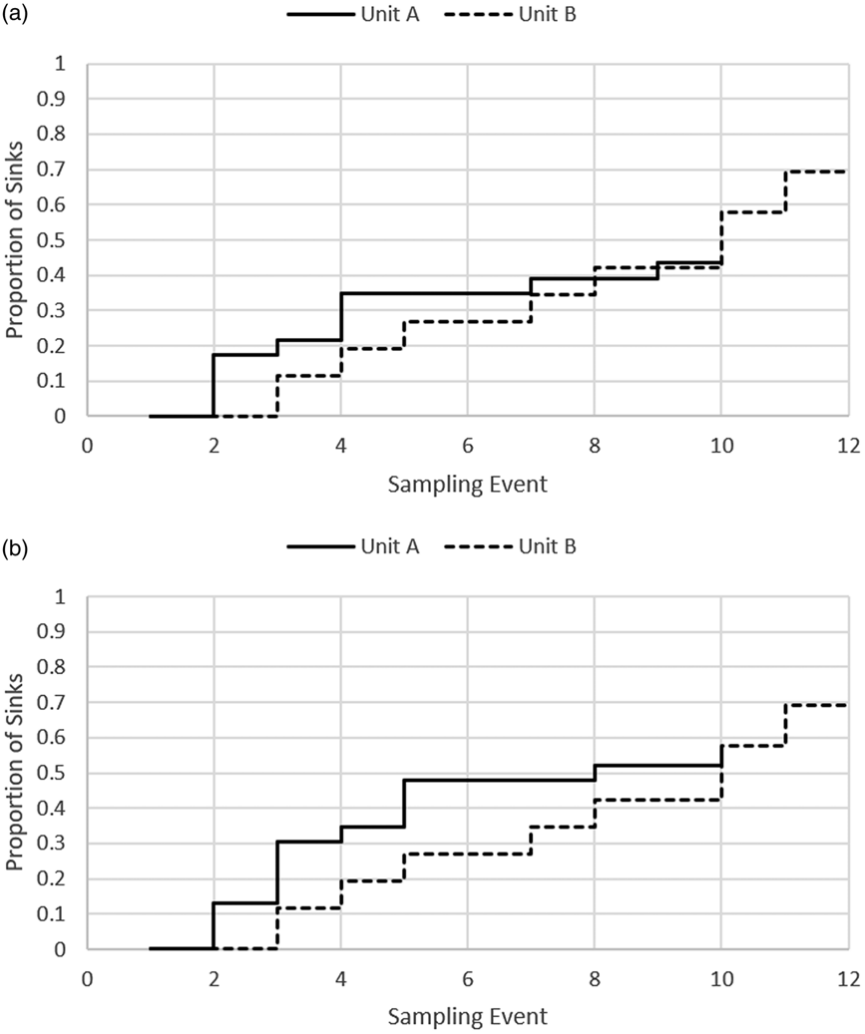
(a) Time to sink conversion event (SCE) with carbapenem-resistant organisms in study sinks. (b) Time to SCE with carbapenemase-producing carbapenem-resistant Enterobacterales in study sinks.

We observed 35 SCEs (73%) overall (Fig. 1). Of 22 remaining sinks in unit A, 9 (41%) became contaminated with CRO by month 10, and all 26 sinks became contaminated with CRO in unit B by month 7. We detected 31 SCEs (63%) with CP-CRE among 49 total study sinks, including 13 (57%) of 23 sinks in unit A and 18 (69%) of 26 in unit B. Despite the infrequent identification of patients with CRE colonization in study units, >50% of the study sinks had newly acquired CP-CRE colonization within a year of the new unit opening (Fig. 1). Among sinks with an identified SCE, the median time to SCE following unit opening was 109 days (IQR, 25–142). The first SCE in unit B took place within 60 days of the unit opening to patients.

Our surveillance strategy allowed us to focus on patients admitted to rooms during the 30 days prior to an SCE in a patient room. Overall, the 1,565 patients residing in rooms 30 days prior to a SCE were generally similar to the 1,251 patients admitted to study units but not in rooms 30 days prior to a SCE, although the latter were more frequently female (Table 2). Notably, the 2 patients identified as colonized with CRE via surveillance testing were, indeed, in rooms within 30 days prior to a SCE.

**Table 2.** Patient and Unit Characteristics During the 30-Day Window before Sink Conversion Events

Characteristic	SCE Associated Patients (N=1,563), No. (%)	Non-SCE Associated Patients (N=1,251), No. (%)	*P* Value
**Patient characteristics**			
Age, median (IQR)	60 (45–71)	61 (48–72)	.91
**Sex**			
Female	720 (46)	641 (51)	.004
Male	841 (54)	600 (48)	
Not Reported/Unknown	4 (0)	10 (1)	
**Race**			
White	902 (58)	773 (62)	.12
Black or African American	517 (33)	369 (29)	
Asian	32 (2)	29 (2)	
Other	70 (4)	42 (3)	
Not Reported/Unknown	44 (3)	38 (3)	
**Unit characteristics**			
Length of stay, median (IQR), d	7 (3–16)	6 (2–15)	.76
**Positive bacteriological cultures, total**	2951	1,637	
Blood	396 (13)	234 (14)	.08
Urine	472 (16)	300 (18)	
Tissue	306 (10)	193 (12)	
Respiratory	81 (3)	42 (3)	
Cerebrospinal fluid	491 (17)	234 (14)	
Other	1205 (41)	634 (39)	
**Positive CRE surveillance cultures, total**	2 (1)	0 (0)	.20
KPC positive	2 (100)	0	
NDM-1 positive	0 (0)	0	
**Patient primary diagnosis and ICD code**			
Malignant neoplasm of frontal lobe (C71.1)	63 (4)	36 (3)	.14
Benign neoplasm of cerebral meninges (D32.0)	60 (4)	62 (5)	
Malignant neoplasm of temporal lobe (C71.2)	53 (3)	26 (2)	
Sepsis, unspecified organism (A41.9)	36 (2)	19 (2)	
Secondary malignant neoplasm of brain (C79.31)	48 (3)	46 (4)	
Trauma subdural hematoma with locatlization of unspecified duration, inititial encounter (S06.5X9A)	29 (2)	21 (2)	
Nontraumatic intracerebral hemorrhage, intraventricular (I61.5)	29 (2)	26 (2)	
Cerebral aneurysm, nonruptured (I67.1)	37 (2)	29 (2)	
Malignant neoplasm of brain, unspecified (C71.9)	30 (2)	17 (1)	
Localization-related (focal) (partial) symptomatic epilepsy and epileptic syndromes with complex partial seizures, intractable, without status epilepticus (G40.219)	31 (2)	5 (1)	
Other	1,149 (73)	964 (77)	

Note. SCE, IQR, CRE, KPC, *Klebsiella pneumoniae* carbapenemase–producing bacteria; NDM-1, New Delhi metallo β lactamase-1; ICD, *International Classification of Diseases*.

### Epidemiologically important pathogens and CRE genes

Overall, 299 CROs were recovered from sink samples; 151 (51%) were *Enterobacter cloacae* complex and 102 (34%) were *Pseudomonas aeruginosa* (Table 3). Although *Enterobacter* spp were the most commonly identified in unit B, *Klebsiella* spp were the most commonly identified CROs in unit A.

**Table 3.** Carbapenem-Resistant Organisms (CROs) Identified in Sink Samples by Unit, Species, and Carbapenemase Gene Presence

Organism	Overall No. (%)	Unit A No. (%)	Unit B No. (%)
**CROs in sink samples**	299	32	267
* Acinetobacter* spp	6 (2)	4 (13)	2 (1)
* Citrobacter* spp	12 (4)	6 (19)	6 (2)
* Enterobacter* spp	151 (51)	5 (16)	146 (55)
* Escherichia coli*	1 (0)	0 (0)	1 (0)
* Klebsiella* spp	27 (9)	14 (44)	13 (5)
* Pseudomonas aeruginosa*	102 (34)	3 (9)	99 (37)
**CROs harboring carbapenemase genes** ^**a**^	42 (16)	9 (28)	33 (12)
* Acinetobacter* spp	0 (0)	0 (0)	0 (0)
* Citrobacter* spp	8 (19)	2 (22)	6 (18)
* Enterobacter* spp	22 (52)	1 (11)	21 (64)
* Escherichia coli*	0 (0)	0 (0)	0 (0)
* Klebsiella* spp	10 (24)	6 (67)	4 (12)
* Pseudomonas aeruginosa*	2 (5)	0 (0)	2 (6)
**Non-EIP harboring carbapenemase genes**	18	12	6
* Comamonas testosteroni*	1 (6)	0 (0)	1 (17)
* Delftia acidovorans*	7 (39)	7 (58)	0 (0)
* Pseudomonas* spp (sans *aeruginosa*)	10 (56)	5 (42)	5 (83)

a Isolates with phenotypic carbapenem resistance were tested for presence of 5 genes (KPC, NDM-1, OXA-48, IMP and VIM)

Of the 299 CROs recovered, 42 (14%) had at least 1 carbapenemase gene; 9 (28%) of 32 in unit A and 33 (12%) of 267 in unit B. The majority of recovered CROs harboring carbapenemase genes were *Enterobacter cloacae* complex [22 (52%) of 42]. Additionally, 18 non-EIP species harboring carbapenemase genes were identified, including 10 *Pseudomonas* spp (not *P. aeruginosa*) and 7 *Delftia acidovorans*. Among all bacteria with an identifiable carbapenemase gene (Table 4), the most common gene was KPC (60%), followed by New Delhi metallo-β-lactamase-1 (NDM-1) (28%) and IMP (active against imipenem; imipenemase) (12%).

**Table 4.** Carbapenemase Gene by Study Pathogen

Pathogen	Total (N=60), No. (%)	KPC (N=36), No. (%)	NDM-1 (N=17), No. (%)	IMP (N=7), No. (%)
**EIP**				
Total	45 (75)	31 (86)	14 (82)	0 (0)
* Acinetobacter* spp	0 (0)	0 (0)	0 (0)	0 (0)
* Citrobacter* spp	9 (20)	7 (23)	2 (14)	0 (0)
* Enterobacter* spp	22 (49)	14 (45)	8 (57)	0 (0)
* Escherichia coli*	0 (0)	0 (0)	0 (0)	0 (0)
* Klebsiella* spp	11 (24)	8 (26)	3 (21)	0 (0)
* Pseudomonas aeruginosa*	3 (7)	2 (6)	1 (7)	0 (0)
**Non-EIP**				
Total	15 (25)	5 (14)	3 (18)	7 (100)
* Comamonas testosteroni*	1 (7)	1 (20)	0 (0)	0 (0)
* Delftia acidovorans*	8 (53)	0 (0)	1 (33)	7 (100)
* Pseudomonas* spp (sans *aeruginosa*)	6 (40)	4 (80)	2 (67)	0 (0)

Note. EIP, epidemiologically important pathogen; KPC, *Klebsiella pneumoniae* carbapenemase–producing isolates; NDM-1, New Delhi metallo-β-lactamase-1; IMP, active against imipenem (imipenemase).

**Table 5.** Gantt Chart: First Isolation of Carbapenem-Resistant Organisms From Study Sinks, Per Species

Year	2020				2021											2022						
Sample	1	2	3	4	5	6	7	8	9	10	11	12	13	14	15	16	17	18	19	20	21	22
Unit	Unit A											Unit B^a^										
Rm																						
1		**DA, PS**	AB									ECC			**ECC**							
2														ECC	**CF**	**ECC**						
3												ECC					PA					
4														ECC					PA			KP
5				KP	PA									**PS**			PA	ECC				
6					**PS**							ECC		PA								
7	**ECC**					KP						ECC			PA							
8		**DA**		PA									ECC		PA							
9	**DA**											ECC		PA								
10					PA										KP, PA	ECC	**CF**					
11		**PS**													PA				PA			
12										AB			ECC	PA				**PS**, PA	EC			**ECC**
13	**PS**, ECC											ECC			PA							**CF, ECC**
14									KP	AB		ECC		PA							**ECC**	
15	AB			CF										ECC	PA			**KP**				
16																	PA				ECC	
17							**DA**							**ECC**, PA								
18													ECC				PA	**PS**, KA				**ECC**
19		**DA**											ECC	PA							KP	**ECC**
20												ECC, PA									**ECC**	
21	ECC		X	**KP**								AB, PA	**ECC**	KO	**PA**			**CF**		**KO**		**PS**
22		PA										AB, ECC		PA							**KP**	
23															PA				ECC			**CF**
24										PA		ECC, PA								PA		
25			**DA**									ECC, PA	**PS**		**CF**							
26		ECC										ECC	**ECC**	**PA**								

Note. AB, *Acinetobacter* spp; CF, *Citrobacter freundii*; DA, *Delftia acidovorans*; ECC, *Enterobacter cloacae* complex; PA, *Pseudomonas aeruginosa*; PS, *Pseudomonas* spp; KO, *Klebsiella oxytoca*; KP, *Klebsiella pneumoniae*; KPC, *Klebsiella pneumoniae* carbapenemase (KPC)-producing bacteria; X, Patient sample positive for KPC-producing *K. pneumoniae*. Bold indicates the presence of carbapenemase gene.

a The new unit opened just after sample 10 was obtained.

No carbapenemase genes were detected in *E. coli* or *Acinetobacter* spp that were identified in sink samples. Of 630 unique-patient microbiological cultures, we removed repeated positive cultures. These cultures were obtained through routine clinical activity, and most were negative. The most common EIPs isolated were *E. coli* (6%), *Klebsiella* spp (2%), and *P. aeruginosa* (1%).

Additionally, 1 study room in each unit was designed and available for patient use; however, these rooms were solely used for medical equipment storage and as a staff break room throughout the study period. Notably, sinks in both study rooms became contaminated with CROs in 86 and 165 days, respectively, following the unit opening.

### Secondary analysis results

The first case of carbapenem-resistant Enterobacterales (CRE) on study units was a 53-year-old woman admitted on December 4, 2020, for sepsis following a urologic procedure. She had no prior CRE infections, but her urine culture at admission grew KPC-producing *K. pneumoniae* (KPC-KP) (Table 5).

In the patient’s room (last checked on November 9, 2020), 3 prior sink samples were negative for KPC-KP. However, on December 7, KPC-KP was detected on the sink’s drain cover, and on December 9, additional environmental samples were taken with sponges from other room surfaces including the patient’s bed, the clinician’s in-room computer, and the medical preparation area. KPC-KP was detected on the computer keyboard and bedrails.

**Figure 2.**
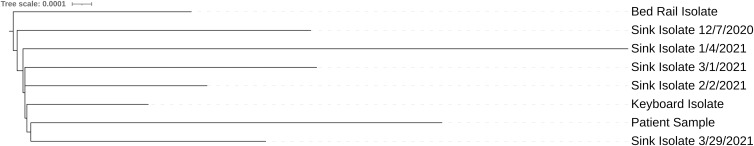
Whole-genome sequencing (WGS) dendrogram of patient, sink and environment *Klebsiella pneumoniae* carbapenemase–producing *Klebsiella pneumoniae* (KPC-KP) isolates.

The patient was discharged from this room December 13, 2020, and the room underwent enhanced terminal room cleaning including UV-C light. On the 4 following routine sink samplings, KPC-KP was recovered again in the p-trap of the index patient’s room sink (Supplementary Fig. 1 online). All isolates were identified as ST-258 and contained the KPC gene. Notably, KPC-KP was not detected in the environment in the same fomites sampled previously following terminal disinfection. Subsequently, short-read WGS confirmed that all 8 isolates were highly related; Newick values were all <0.004 (Fig. 2).

On April 5, 2021, an intervention was implemented to eradicate KPC-KP from the sink, detailed in the Supplementary Material (online). After the intervention, environmental samples were negative for KPC-KP, with weekly monitoring until September 1, 2021, and a final check on November 1, 2022. Postintervention samples revealed typical contaminants but no KPC-KP.

## Discussion

Sinks in healthcare settings often contain pathogenic bacteria, including bacteria harboring genes that confer high-level resistance to multiple antibiotics. These sinks have been identified as the source of in-hospital transmission to patients and outbreaks of infection, leading to the recognition that a sink, often situated less than 1 meter from a patient, can serve as an important reservoir for multidrug-resistant pathogens, such as CROs.^5–13^ However, the timing, source, and frequency of sink contamination with CROs are not completely understood. To our knowledge, our study is the first to prospectively evaluate how quickly and to what extent in-room sinks become contaminated with CRO in a new hospital bed tower. In our study, CRO contamination started within 2 months of unit opening; overall, >70% of in-room sinks were contaminated with 1 or more CRO and >60% were contaminated with 1 or more CP-CRE within a year of opening.

CRO sink contamination increased over time, but the source of increasing contamination was not clear. On surveillance testing, 2 patients were identified as having colonization, and one of the sinks of these rooms was contaminated with the same CRO thereafter. No patient had CRO infection. Thus, no definitive source was otherwise identified for the 30 other SCEs.

Although surveillance for CRE was routinely performed in study units, most patients were not screened, likely due to short length of stays in study units. Thus, patients with undetected CRO colonization could have contaminated other sinks. As outlined above, 1 room in each unit never housed a patient and was instead used as an employee break room. Nonetheless, SCEs occurred in this unique room type in both units. Other potential sources of contamination could be employee colonization, cross colonization between sinks with shared premise plumbing, or healthcare personnel hand contamination in one room and use of sinks in an adjacent room.^9^ Additionally, we detected differences between units, such as sink colonization with CRO, which was notably lower in unit A (60%) compared to unit B (100%), and most *P. aeruginosa* were recovered in unit B. However, we are unaware of any concrete reasons for these differences.

For our secondary analysis, in a new hospital bed tower with no prior evidence of CRE-positive patients, the first identified case of a CRE (KPC-KP) in a patient resulted in environmental contamination of the room after only 3 days of hospitalization. Contamination of the in-room sink drain known to be CRE negative persisted for 4 months before our disinfection intervention. Our intervention was successful in eliminating KPC-KP from the sink for 149 days. We hypothesize that our intervention was successful because it was on a sink in a new hospital bed tower without known previous colonization. The rapid response likely prevented colonization of plumbing beyond the sink.

However, our experience and data support the idea of monitoring sink colonization in CRE patient rooms following their detection and intervening to prevent colonization. The intervention was straightforward, inexpensive, and did not require significant time to perform nor special equipment. Additional research is needed to evaluate the efficacy and durability of the decontamination intervention on sinks with varied ages of biofilms because sink drain decontamination efforts are not often successful.^15^

The presence of CRO in sinks in hospitals should be expected. Proper hand hygiene, in fact, should lead to the removal of CRO and other multidrug-resistant organisms (MDROs) from hands to sinks. However, other activities and sink uses may promote growth and persistence of these organisms, including disposal of body fluids, IV fluids, and tube feeds.^3,9^ Regardless of how the organisms contaminate these sinks, our data and data from others confirm that these contaminated sinks represent an important source for potential CRO exposure in acute-care hospital rooms, often harbor multiple species with carbapenem resistance from multiple mechanisms, and are difficult to eradicate.

For example, De Geyter et al^12^ investigated CRE contamination of sinks in an ICU during an outbreak with *Citrobacter freundii* harboring OXA-48. These investigators reported that every isolation room, except one, was contaminated with CRE including *Citrobacter* spp similar to the outbreak strain but also other species, including *Enterobacter cloacae complex* and *Klebsiella* spp with KPC, NDM-1, and/or OXA-48 genes.^12^ These investigators replaced sink siphons in the unit and implemented more rigorous infection prevention practices, including a more rigorous sink disinfection strategy, increased emphasis on hand hygiene, and education on using the sinks only for hand washing. Despite these efforts, 9 (28%) of 32 sinks remained contaminated with CRE. Similarly, Franco et al^13^ sampled sinks in 2 units every 3-weeks for a 12-week period and evaluated growth of CRE and *Pseudomonas* spp in patient-care sinks as well as sinks used by healthcare personnel. CROs, including *Enterobacter cloacae* complex, *Klebsiella* spp and *Citrobacter* spp were more commonly encountered in patient-room sinks. *P. aeruginosa* was frequently detected in both sink types but was more likely to harbor resistance genes in patient care sinks.

Our findings suggest that CROs were introduced into sinks of a newly constructed unit only after patient care began in that unit and increased over time with ongoing clinical activity. These findings differ from those of Sukhum et al,^14^ who found no increase in the number of antimicrobial-resistant organisms within in-room patient sinks in a newly constructed ICU before and after patients were treated in the unit. This discordance could be related to differences in study design. Our study targeted carbapenem-resistant gram-negative organisms via selective growth media, whereas Sukhum et al^14^ included all antimicrobial-resistant organisms, such as gram-positive organisms, intrinsically resistant *Stenotrophomonas* spp, *Candida* spp, among others.^14^

Our study had several limitations. First, culture-based sink surveillance may underestimate the presence of CRO. Various factors related to sink use may affect these results, including the presence of other fluids such as soap or clinical biowaste. In contrast, we attempted to increase our likelihood of identifying contaminating organisms by specifically agitating the p-trap fluid to retrieve biofilm. Metagenomic evaluation may improve our ability to identify the emergence of resistance in these settings. Second, active surveillance performed in the units likely underestimated the prevalence of CRO in patients. As noted, sampling was limited to ∼30% of patients. In addition, surveillance testing was limited to CRE and did not evaluate for other CROs. Third, our study did not include molecular evaluation of isolates; thus, we were unable to confirm similarity of isolates in different locations. Finally, we only assessed sink contamination once a month, which likely affected the ability to capture sink contamination granularity during the study.

Our findings have demonstrated the rapid and cumulative contamination of sinks by CRO in 2 newly constructed ICUs. Our results, paired with growing literature, support in-room hospital sinks as an important reservoir of CRO, and they emphasize the need for infection prevention strategies to mitigate contamination of surfaces from sinks (eg, splash guards) as well as the development of novel strategies to eliminate CRO from sinks. Future studies should evaluate the utility of routine surveillance for CRO contamination in sinks and should compare primary versus secondary prevention strategies to reduce risk of patient transmission and harm. Our findings also suggest that the sources and causes of sink contamination are still not well understood. Molecular and metagenomic techniques may provide additional information and should be included in future studies. In summary, our results indicate that sink contamination with CRO in a new hospital bed tower is rapid, has a cumulative effect, and is arguably inevitable.

## Supplementary material

For supplementary material accompanying this paper visit https://doi.org/10.1017/ice.2023.289. click here to view supplementary material
