# Supplementary material for: Identification of carbapenem-resistant organism (CRO) contamination of in-room sinks in intensive care units in a new hospital bed tower
Source: Infect Control Hosp Epidemiol. 2024 Jan 19;45(3):302–9. doi: 10.1017/ice.2023.289 (PMC10933507; doi:10.1017/ice.2023.289)
Supplement: Supplementary file 1 [file S0899823X23002891sup.zip › S0899823X23002891sup001.docx]

Supplemental Methods

*Microbiological Methods*

The top of bowl and drain cover samples were obtained with cellulose sponges premoistened with neutralizing buffer (3M, Saint Paul, Minnesota) using the sponge and stomacher technique.^1^ Sponges were placed in stomacher bags with 45 mL phosphate-buffered saline with 1% Tween 20 and homogenized for 60 seconds at 260 revolutions per minute (RPM). Homogenates were then centrifuged at 3200 RPM for 15 minutes, and all but approximately 5 mL of the resulting supernatant was discarded. Then, each sample was rehomogenized via vortex. A total of 200 μL of the final homogenate was plated onto HardyCHROM CRE media (Hardy Diagnostics, Santa Maria, CA) and incubated at 37°C for 24 hours. The p-trap sample was obtained by feeding sterile tubing attached to a sterile 50 mL syringe down the drain into the p-trap fluid, agitating the fluid by pulling and pushing the syringe plunger aggressively to slough portions of biofilms into the fluid, and then using the syringe to collect 50 mL of agitated p-trap fluid.^2^ This 50 ml was vortexed, serially diluted and 50 μL was plated onto HardyCHROM CRE media (Hardy Diagnostics, Santa Maria, CA) and incubated at 37°C for 24 hours.

All growth on selective media was subcultured to MacConkey agar to remove potential Gram positives as mentioned in the limitations of the media’s instructions for use. Gram negative growth was considered carbapenem-resistant (CR) and carbapenemase gene presence was assessed via PCR with primers designed for KPC, NDM-1, OXA-48, IMP, and VIM.^3^ Isolate species in the primary analysis were confirmed using 16S rRNA gene sequencing.^4,5^ CRO were defined as epidemiologically important pathogens (EIP) that grew on study media. EIP were chosen based on a combination of clinical relevance and the study’s chosen selective media’s capabilities and were defined as *Acinetobacter spp., Citrobacter spp., Enterobacter spp., Escherichia coli, Klebsiella spp., and Pseudomonas aeruginosa;* other identified organisms were considered non-EIP including *Stenotrophomonas spp.* and *Serratia spp.*.

*Secondary analysis methods*

For this secondary analysis, species were confirmed via MALDI-ToF, antimicrobial resistance genes were confirmed via PCR, as above, and relatedness was assessed via Multi-locus sequence typing (MLST) and whole genome sequencing (WGS).^6^ For WGS, isolates were compared using mashtree and dendrograms were generated as previously described.^7^

Supplemental Results

Sink intervention: 1) The colonized p-trap was removed, 2) the wall outlet the p-trap drains into and the bottom of the sink drain were mechanically cleaned with a scrub brush drenched with a hospital grade detergent, 3) the bottom of the sink drain was capped and the drain was filled with a 10% sodium hypochlorite bleach solution and allowed to sit for 15 minutes 4) a new p-trap was installed, 5) the p-trap was refilled with tap water.

References

1. International Organization for Standardization I. Sterilization of medical devices — microbiological methods Part 1. *ISO Stand*. 1995;11737-1.

2. Franco LC, Tanner W, Ganim C, Davy T, Edwards J, Donlan R. A microbiological survey of handwashing sinks in the hospital built environment reveals differences in patient room and healthcare personnel sinks. *Sci Rep*. 2020;10(1):8234. doi:10.1038/s41598-020-65052-7

3. Solanki R, Vanjari L, Subramanian S, B A, E N, Lakshmi V. Comparative Evaluation of Multiplex PCR and Routine Laboratory Phenotypic Methods for Detection of Carbapenemases among Gram Negative Bacilli. *J Clin Diagn Res*. 2014;8(12):DC23-6. doi:10.7860/JCDR/2014/10794.5322

4. Janda JM, Abbott SL. 16S rRNA gene sequencing for bacterial identification in the diagnostic laboratory: pluses, perils, and pitfalls. *J Clin Microbiol*. 2007;45(9):2761-2764. doi:10.1128/JCM.01228-07

5. Srinivasan R, Karaoz U, Volegova M, et al. Use of 16S rRNA gene for identification of a broad range of clinically relevant bacterial pathogens. *PLoS One*. 2015;10(2):e0117617. doi:10.1371/journal.pone.0117617

6. Diancourt L, Passet V, Verhoef J, Grimont PAD, Brisse S. Multilocus sequence typing of Klebsiella pneumoniae nosocomial isolates. *J Clin Microbiol*. 2005;43(8):4178-4182. doi:10.1128/JCM.43.8.4178-4182.2005

7. Katz L, Griswold T, Morrison S, et al. Mashtree: a rapid comparison of whole genome sequence files. *J Open Source Softw*. 2019;4(44):1762. doi:10.21105/joss.01762
