# Supplementary figures and images for: Identification of carbapenem-resistant organism (CRO) contamination of in-room sinks in intensive care units in a new hospital bed tower
Source: Infect Control Hosp Epidemiol. 2024 Jan 19;45(3):302–9. doi: 10.1017/ice.2023.289 (PMC10933507; doi:10.1017/ice.2023.289)

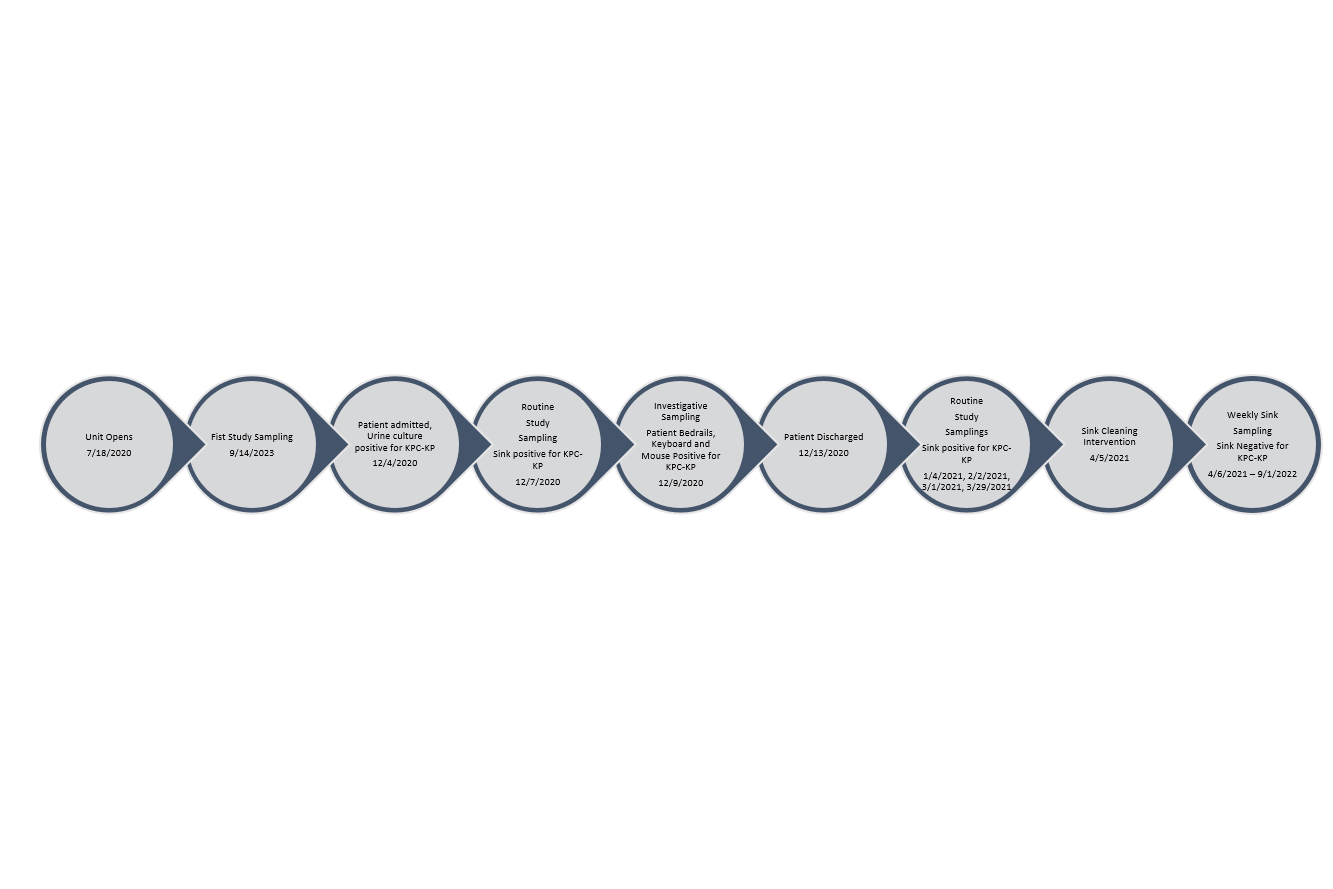

Supplement: Supplementary file 1 [file S0899823X23002891sup.zip › S0899823X23002891sup002.tif]
